# Comparing temperature-related mortality impacts of cool roofs in winter and summer in a highly urbanized European region for present and future climate

**DOI:** 10.1016/j.envint.2021.106606

**Published:** 2021-09

**Authors:** Helen L. Macintyre, Clare Heaviside, Xiaoming Cai, Revati Phalkey

**Affiliations:** a Climate Change and Health Group, Centre for Radiation Chemical and Environmental Hazards, Public Health England, Chilton, Oxon OX11 0RQ, UK; b School of Geography, Earth and Environmental Sciences, University of Birmingham, Edgbaston, Birmingham B15 2TT, UK; c Institute for Environmental Design and Engineering, University College London, Central House, 14 Woburn Place, London WC1H 0NN, UK; d Division of Epidemiology and Public Health, School of Medicine, University of Nottingham City Hospital, Hucknall Road, NG51PB Nottingham, UK; e Heidelberg Institute for Global Health, University of Heidelberg, Im Neuenheimer Feld 130.3 69120 Heidelberg, Germany

**Keywords:** Urban health, Temperature exposure, Climate change, Cool roofs, Adaptation and mitigation, WRF

## Abstract

Human health can be negatively impacted by hot or cold weather, which often exacerbates respiratory or cardiovascular conditions and increases the risk of mortality. Urban populations are at particular increased risk of effects from heat due to the Urban Heat Island (UHI) effect (higher urban temperatures compared with rural ones). This has led to extensive investigation of the summertime UHI, its impacts on health, and also the consideration of interventions such as reflective ‘cool’ roofs to help reduce summertime overheating effects. However, interventions aimed at limiting summer heat are rarely evaluated for their effects in wintertime, and thus their overall annual net impact on temperature-related health effects are poorly understood.

In this study we use a regional weather model to simulate the winter 2009/10 period for an urbanized region of the UK (Birmingham and the West Midlands), and use a health impact assessment to estimate the impact of reflective ‘cool’ roofs (an intervention usually aimed at reducing the UHI in summer) on cold-related mortality in winter. Cool roofs have been shown to be effective at reducing maximum temperatures during summertime. In contrast to the summer, we find that cool roofs have a minimal effect on ambient air temperatures in winter. Although the UHI in summertime can increase heat-related mortality, the wintertime UHI can have benefits to health, through avoided cold-related mortality. Our results highlight the potential annual net health benefits of implementing cool roofs to reduce temperature-related mortality in summer, without reducing the protective UHI effect in winter.

Further, we suggest that benefits of cool roofs may increase in future, with a doubling of the number of heat-related deaths avoided by the 2080s (RCP8.5) compared to summer 2006, and with insignificant changes in the impact of cool-roofs on cold-related mortality. These results further support reflective ‘cool’ roof implementation strategies as effective interventions to protect health, both today and in future.

## Highlights

• Urban heat interventions generally aim to reduce heat-related effects in summer.
• Cool roofs have a larger impact on temperature in summer than in winter.
• In winter cool roofs have negligible impacts on cold-related mortality.
• Cool roofs have net benefits in reducing annual temperature related mortality.

## 1 Introduction

Hot or cold weather can negatively impact human health, potentially exacerbating conditions such as respiratory or cardiovascular diseases, and leading to increased risk of hospitalization and death (Basu, 2009, Gómez-Acebo et al., 2013, Guo et al., 2014). Projections suggest that UK mean air temperatures could increase by between 0.7 °C and 4.2 °C in winter, and 0.9 °C to 5.4 °C in summer by 2070 (unmitigated emissions, RCP8.5), with weather extremes also projected to become more frequent and intense (Lowe et al., 2018). With climate change, it is estimated that heat-related mortality could increase from ~2000 to ~7000 deaths per year by 2050 in the UK (for a medium emissions scenario), while there is relatively little reduction in cold-related mortality, partly due to population growth and ageing (Hajat et al., 2014, HPA, 2012). However, in the UK, cold effects on health currently outweigh those from heat, with estimates suggesting for the 2000s that cold-related deaths were around 41,000 annually, compared with around 2,000 heat-related deaths (Hajat et al., 2014). Studies have shown mixed results on whether the UK population might adapt to heat (Arbuthnott et al., 2016, Milojevic et al., 2016).

Temperature may also be influenced by the Urban Heat Island effect (UHI), where urban materials such as buildings and roads can lead to elevated temperatures in towns and cities compared with rural areas. The UHI has been shown to contribute to negative heat-health outcomes (Heaviside et al., 2017), with the summer UHI associated with increases in heat-related mortality, and effects in winter being less clear, though studies suggest a protective effect on cold-related mortality; for the West Midlands, a heavily urbanised area of the UK, it has been shown that ~40% of heat-related mortality could be attributed to the UHI intensity during summer 2006, and up to 50% during the heatwave of 2003, with increasing impacts on heat-related mortality in future (Macintyre and Heaviside, 2019, Heaviside et al., 2016). The winter UHI has received far less attention than the summer UHI, but has been shown to potentially protect against up to 15% of cold-related mortality in a cold winter (UK, 2009–2010), with the future impacts showing little change (Macintyre et al., 2021).

Due to the high fraction of the population living in urban areas (82% in the UK, (ONS, 2011)), climate change, and the UHI, urban populations are at particular risk from heat. As such, interventions to limit urban heat (such as reflective ‘cool’ roofs, or urban green infrastructure) have been proposed as measures to help reduce impacts from heat in urban areas (Besir and Cuce, 2018, Li et al., 2014, Silva et al., 2010). Such interventions in the built environment would typically be in place year-round, yet the effects are seldom studied outside of the summer period, and thus their impacts over the year are poorly understood. It is clear that the UHI may have a protective effect against cold-related mortality in wintertime in the UK, although it is unclear how urban heat mitigation measures aimed at reducing summer heat, such as reflective ‘cool’ roofs, might alter this protective effect. Therefore, understanding of the wintertime impacts of such interventions is required to avoid maladaptation. This motivates a study on the impacts of interventions for urban heat on both heat- and cold-related mortality, including how this might change as climate changes.

Studies suggest that the average intensity of the UHI in summer is around 1–4 °C, being slightly larger than winter UHIs (1–3 °C), and with a larger diurnal cycle in summer (Gedzelman et al., 2003, Imhoff et al., 2010, Kłysik and Fortuniak, 1999, Runnalls and Oke, 2000, Yang and Bou-Zeid, 2018). In summer, higher sun angles and longer days result in more solar radiation being absorbed and subsequently released from urban structures. This leads to a more pronounced diurnal cycle in the summer UHI, with the UHI being particularly strong at night-time (Santamouris, 2015). In the UK, the UHI for a number of cities has been estimated at 1–2 °C in winter, and 2–3 °C in summer (Kershaw et al., 2010). For Birmingham and the West Midlands, the winter UHI, previously estimated at 2.3 °C, and of similar magnitude to the summer UHI, has a protective effect on health in winter, through reduced cold-related mortality. However, studies suggest that heat-related mortality associated with the UHI in summer could increase significantly with climate change, whereas the change in cold-related mortality will be modest (Heaviside et al., 2016, Macintyre et al., 2021).

Mitigation and adaptation measures in the built environment designed to reduce summer temperatures in cities also have the potential to reduce energy consumption for cooling demand as well as improve health (Kolokotroni et al., 2012, van Ruijven et al., 2019), potentially contributing to net zero emissions targets. Measures to reduce urban heat mostly focus on modifying the urban fabric of cities (as this is often the main driver of the UHI), including improved urban planning, modifying building materials, increasing green/blue space, and reflective ‘cool’ facades with higher albedo (U.S. Environmental Protection Agency, 2008). Rooftops are an effective area to target for cooling interventions as they typically receive the greatest incident solar radiation and are often the largest area of internal heat gain to buildings; reflective ‘cool’ roofs have a higher albedo (reflectivity) to reflect solar radiation, which in turn can help reduce urban temperatures (Li et al., 2014, Oleson et al., 2010). Simulations of such roofing interventions in cities in the US and Europe have shown peak reductions on summertime local air temperatures of around 1–3 °C, with effects increasing roughly linearly with coverage (Li et al., 2014, Morini et al., 2016, Morini et al., 2018, Smith and Roebber, 2011, Vahmani et al., 2016). A wintertime modelling study on US cities found cool roofs reduced 2 m air temperatures by 1.4 °C during the day (Yang and Bou-Zeid, 2018). Modelling studies on London suggested that cool roofs could reduce maximum air temperature by 1 °C in summer (Virk et al., 2015), and indoor median summer temperatures by 0.6 °C (Taylor et al., 2018); overall annual heating energy demand increased slightly (4.1%) for current climate (Taylor et al., 2018), however, for a 2050 climate scenario, cool roofs resulted in a net reduction in annual energy use (Virk et al., 2015), and have been found to be preferable to natural ventilation, as there was a lower winter penalty for heating demand (Kolokotroni et al., 2013).

A few studies have examined the annual (as opposed to summer only) health impacts of building interventions for reducing urban heat, finding reductions in heat-related mortality (Stone et al., 2014, Susca, 2012), however impacts on cold-related mortality were not considered. A recent study considered the health impacts in summer and winter of cool and green roofs over an urbanized area in the U.S., finding that cool roofs reduced heat exposure in summer, but could potentially exacerbate cold exposure in winter; green roofs had smaller beneficial impacts on summer heat, but almost no negative impact in winter (He et al., 2020). Previous work estimated the impact of cool roofs in a UK city could potentially offset up to 25% of heat-related mortality associated with the UHI during heatwave periods, and 18% over a summer season (Macintyre and Heaviside, 2019). However, there is a clear gap in understanding related to how such interventions will perform in winter, including a potential reduction in any protective health effect of the wintertime UHI. It is important to assess any co-benefits/unintended consequences of measures aimed at limiting summer urban heat, and how these effects and impacts may change with climate change.

In order to determine potential wintertime effects of UHI mitigation measures implemented primarily to reduce hot summer temperatures, we use a regional weather forecasting model to simulate the effect of reflective ‘cool’ roofs on urban temperatures in winter, and quantify the subsequent impact on temperature-related mortality (cold-related deaths avoided) using a health impact assessment based on application of an existing exposure-response relationship (Section 2.2). We also include climate change projections to investigate how the impacts of such interventions may change in future.

## 2 Methods

To determine the impact of cool roofs on ambient temperature, we simulate 2 m ambient air temperature across the West Midlands using a regional configuration of a mesoscale meteorological model (Section 2.1). The output from this model gives us high-resolution gridded daily temperature which is used as input to the Health Impact Assessment (HIA) to calculate temperature-related mortality. To quantify the impact of cool roofs on urban air temperatures, we adjusted roof albedo in the urban model during a winter period (mid-November 2009 to the end of March 2010). This winter was chosen as a particularly cold winter to compare with the impacts of cool roofs examined for hot summers in previous studies (Macintyre and Heaviside, 2019). Our HIA is based on application of an existing exposure-response relationship derived for the population in question in the West Midlands of the UK (Vardoulakis et al., 2014) to the temperature (exposure) data; the exposure response coefficient we use quantifies the relationship between daily ambient temperature and increased risk of all-cause mortality. Our Health Impact Assessment (HIA) assesses the potential impact cool roofs on cold-related mortality in winter, and we compare this with previous results for cool roofs in summer. Finally, we use the latest climate change projection data for the UK to see how the impact of interventions might change in a future scenario.

### 2.1 Urban temperature modelling

In order to estimate the impacts of interventions on temperature and health, temperatures must be compared between scenarios with and without the intervention. Modelling is a useful tool for this as we can alter the properties of the built environment in a model to see what theoretical change in temperature might occur, while holding all other properties constant in order to isolate the impact of the intervention alone. We use modelled gridded 2 m temperatures as simulated by the WRF (Weather Research and Forecasting) model (Chen et al., 2011), which is a comprehensive analysis model that has previously been used to study the impact of building interventions to modify the UHI at urban and regional scales. To facilitate comparison with a previous study which examined the impact of cool roofs in a summer season (Macintyre and Heaviside, 2019), we use the same model set up as detailed in that study and also in Macintyre et al. (2021). We use WRF model version 3.6.1 and include a multi-layer urban canopy scheme (BEP – the Building Energy Parameterization scheme) configured with 3 separate urban land classifications, specifically for Birmingham and the West Midlands, which allows us to capture the effect of the built environment on the lower atmosphere (Martilli et al., 2002). The model has been previously run and evaluated against available observations for this specific set up and time period; a summary of the model set up, urban category details, and model evaluation statistics are presented in the supplementary materials (Table S1-2). The model was evaluated against hourly data from available Met Office Integrated Data Archive System (MIDAS) (Met Office, 2012) stations in the inner domain, using bilinear interpolation from the nearest four grid points. The model is able to capture the diurnal variation in temperature, with correlation being 0.90 or higher, with comparison being slightly better for urban sites (Table S2).

**Fig. 1:**
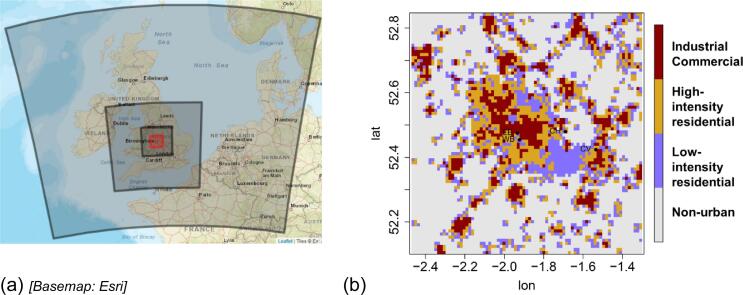
(a) Modelled domains in the WRF simulation. The central (red) box is the innermost domain, which is expanded in (b); (b) Urban categories used in the inner domain; the area covered is ~80 × 80 km. Lettered points refer to observation stations within the domain (refer to Table S2).

We simulated hourly 2 m air temperature across the model domain (Fig. 1a) for 14 November 2009 to 28 February 2010 (first day discarded as spin-up) with detailed categories of urban surfaces included (the ‘URBAN’ simulation). The model domain and urban categories across the inner domain (at 1 km horizontal grid resolution) are shown in Fig. 1. To give a reference for 2 m temperature for a non-urban setting, we ran the same model again, but replaced the urban categories with rural types (the ‘RURAL’ simulation).

To simulate the effect of cool roofs being introduced across the West Midlands, we altered albedo of all roofs in the URBAN set up from the default values (~0.2, Supplementary Table S1a) to a value of 0.7 (‘COOLROOF’) within the BEP scheme (with walls and ground surfaces unchanged). The value 0.7 was chosen as a compromise between the fact that higher albedos are achievable, but reflect the fact that cool roofs may degrade with time (see supplementary material for details for other standard model settings). We quantified the impact of cool roofs in winter by taking the difference in temperature (population-weighted, as described below in Section 2.2) between the URBAN and COOLROOF simulations.

To estimate the impacts of cool roofs on winter and summer UHIs in future climates, we used the central estimate (50th percentile) of seasonal temperature projections from the UKCP18 suite of probabilistic projections over land for RCP8.5 (at 25 km), extracted for the West Midlands region. The values (Table 1) are applied as a simple temperature increment to the hourly modelled values from the WRF model for all scenarios as a sensitivity study for a future climate scenario. Winter values are applied to the model simulations described in 2.1, and summer values are applied to simulated data for 1 June–31 August 2006 using the same modelling set up (described in Macintyre and Heaviside (2019)). To aid comparison of overall temperature-related mortality for each of the seasonal periods, we considered the HIA over the same number of days in each season (14 June–31 August 2006 for summer, and 12 December 2009–28 February 2010 here for winter). Population size and demographics are held constant at present day levels, and daily mortality counts are not adjusted for future scenarios.

**Table 1:** Mean seasonal projected temperature changes from the UKCP18 probabilistic projections (25 km) for RCP8.5. Each time horizon is based on 30-year time slices (2050s is based on 2040–2069 and 2080s is based on 2070–2099), relative to a 1981–2010 baseline. Data is extracted via the Met Office user interface web portal for the West Midlands administrative region (https://ukclimateprojections-ui.metoffice.gov.uk).

	**2050s**	**2080s**
**Summer**	2.22 °C	4.66 °C
**Winter**	1.65 °C	3.01 °C

### 2.2 Health impact calculations.

We estimate the health impacts (mortality) associated with high or low temperatures by combining a published exposure-response coefficient which relates daily mean temperature and the increased risk of mortality, with modelled ambient temperature (Section 2.1), and applying to daily all-cause mortality counts. Effects are calculated based on the difference between ambient temperatures and the relevant thresholds for effects (calculated in the epidemiological study). In the UK, pooled estimates show that for heat effects, mortality increases by 2.5% for every 1 °C above a daily mean threshold temperature of around 18 °C, and for cold effects, there is a 2.0% increase in mortality for every 1 °C below a daily mean threshold of ~12 °C (Hajat et al., 2014, Vardoulakis et al., 2014). We calculated the cold-related mortality over the winter period, under the COOLROOF and URBAN scenarios, by calculating daily cold-related mortality, $M_{i}$, and summing over all days, i, using the following method:

$$M_{i}=D_{i}\times {\mathrm{AF}}_{i}$$

$${\mathrm{AF}}_{i}=\left(\frac{{\mathrm{RR}}_{i}-1}{{\mathrm{RR}}_{i}}\right)$$

$${\mathrm{RR}}_{i}=e^{\left(b{\Delta T}_{i}\right)}$$

where $D_{i}$ is the all-cause mortality count for day i, AF (attributable fraction) is the fraction of daily mortality that can be attributed to the effects of cold exposure, defined by RR, which is the relative risk, depending on b, the slope of the exposure-response relationship, and ${\Delta T}_{i}$ is the difference between the mean daily population-weighted temperature for day i and the threshold temperature for effects. We use the exposure-response coefficient derived in Vardoulakis et al. (2014), for the West Midlands region, which is approximately a 1.8% (95% CI: 1.6%, 2.1%) increase in mortality for every 1 °C decrease in daily mean temperature below the threshold of 11.7 °C (representing the 60th centile daily mean temperature for this region); the daily mean temperature is the mean temperature over that day and the preceding 27 days, representing the longer lag period for cold effects (as opposed to the 0–1 day lag for heat effects (Vardoulakis et al., 2014)), and so health effects are calculated from 12 December 2009 to 28 February 2010. Exposure is calculated based on gridded hourly 2 m air temperature output from the WRF model to calculate daily mean temperatures, and population weighting is by using a gridded 100 m residential population database (ONS, 2011, ONS, 2015). Age-stratified exposure-response coefficients were available for the following age groups: 0–64, 65–74, 75–84, and 85+ years; for age-group population weighted temperature, demographic information at Output Area^1^ (OA) level from the most recent census (ONS, 2011) was combined with the gridded population data above, following the method described in Macintyre et al. (2018). Daily all-cause mortality counts were obtained from the Office for National Statistics. Differences between daily mean population weighted temperatures between the temperature scenario simulations (URBAN, RURAL, COOLROOF), and the estimated total daily cold-related mortality values were tested for significance at the 95% level using a *t*-test (two-sample, testing for equal variances).

## 3 Results

### 3.1 Impact of cool roofs in winter

**Fig. 2:**
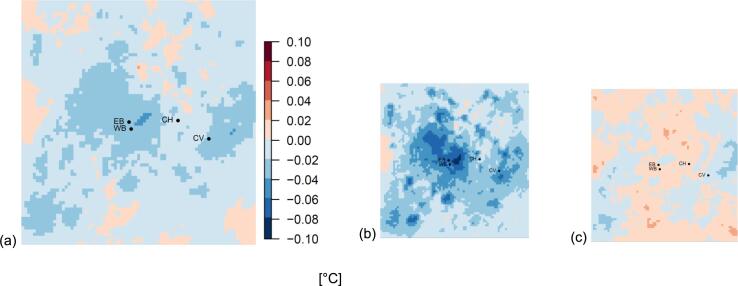
Impact of cool roofs for 15 November 2009 – 28 February 2010 in terms of reduction of 2 m temperature. (a) Average temperature difference for the whole period; (b) daytime average (8am – 8 pm); (c) night-time average (8 pm – 8am). City centre in this figure is −0.04 °C (−0.08 °C daytime, −0.01 °C at night). Letters refer to observation stations (refer to Fig. 1b and Table S2).

The effect of cool roofs (the difference in 2 m temperature between COOLROOF and URBAN simulations) is shown in Fig. 2, with the average over all times (Fig. 2a), day (Fig. 2b) and night time (Fig. 2c) averages.

**Fig. 3:**
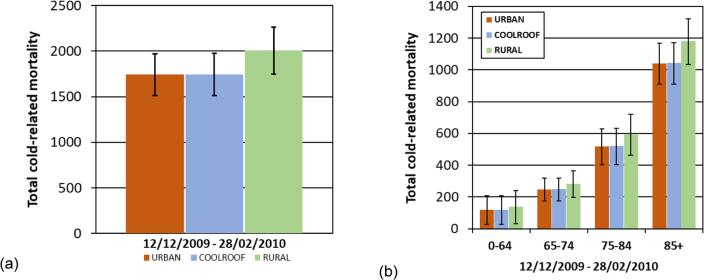
Cold-related mortality for the West Midlands for URBAN, COOLROOF AND RURAL simulations; Total population (a) and age stratified (b). Bars represent 95% confidence intervals on the exposure–response relationship derived by Vardoulakis et al. (2014).

A small cooling effect during the daytime is seen (Fig. 2b), but the effect on mean population-weighted 2 m air temperatures is modest, 0.05 °C (daytime, Table 2), which corresponds to ~3% of the UHI intensity (difference in daily mean population-weighted 2 m temperatures is not statistically significant, *p* = 0.96). Previous simulations of the effect of cool roofs showed a larger effect of cool roofs in summer, being an order of magnitude greater (mean population-weighted temperature difference up to −0.6 °C) in the city centre (Macintyre and Heaviside, 2019), compared with winter values of −0.08 °C here for daytime city centre (Fig. 2). Maximum cooling reached −3.1 °C in the city centre in summer, compared to −0.5 °C and occasionally −0.7 °C in winter (not shown). During summer, the greater solar insolation, higher sun angles, and often less cloud means incoming solar energy is the main driver of the UHI, and thus there is more opportunity for cool roofs to influence the UHI by reflecting sunlight, leading to their larger impact on ambient temperatures in summer rather than winter. Due to this smaller impact of solar insolation on air temperatures in wintertime, other sources of heat such as from inside buildings are likely to have more influence on urban ambient temperatures than in summer. We find that in our study, cool roofs have a negligible effect on cold-related mortality (4 additional cold-related deaths; 0.23% of overall cold-related deaths) in winter, which corresponds to 1% of those avoided due to the UHI (Fig. 3a).

**Table 2:** Temperature statistics for different model simulations for the ~11 week winter period (15 November 2009–28 February 2010). Values are population weighted averages across the whole modelled domain, and broken down for day and night times. Numbers have been rounded from calculations with exact figures.

*Population weighted*	‘URBAN’ run T 2 m (°C)	‘COOLROOF’		UHI (‘URBAN’– ‘RURAL’) (°C)	% of UHI offset by cool roofs
		T 2 m (°C)	ΔT (°C) (‘COOLROOF’– ‘URBAN’)		
Mean	3.28	3.26	−0.024	1.48	1.6%
Day	3.50	3.45	−0.047	1.36	3.5%
Night	3.01	3.01	−0.000	1.54	0.0%

To contextualise our results, for the same winter period and region, we previously showed that the regional population-weighted mean UHI intensity was +1.5 °C (+1.54 °C at night, +1.36 °C during the day), with the mean UHI intensity being highest in the city centre, at +2.4 °C on average over winter, and +2.5 °C at night. In contrast to the typical summer UHI, which tends to be more intense during the night than the day, the UHI was more similar between day and night in winter (Macintyre et al., 2021).

### 3.2 Impacts in the context of climate change

The HIA for the COOLROOF simulation was repeated with the same modelled data as described above, with addition of the temperature increment of projected changes across the West Midlands for a high emissions scenario (RCP8.5), compared to the 1981–2010 baseline (Table 1); for ease of comparison we refer to previous results for the URBAN simulation (Macintyre et al., 2021).

**Fig. 4:**
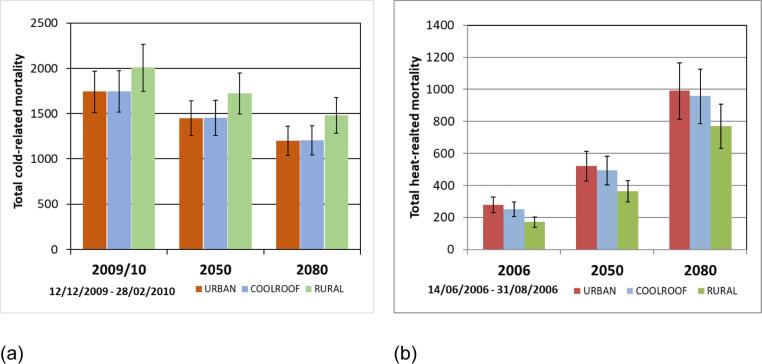
(a) Cold-related mortality and (b) heat-related mortality across the West Midlands for different climate scenarios. 2050s and 2080s 50th percentile from the UKCP18 probabilistic projections (25 km) over land. Error bars show the range related to the 95% confidence intervals on the exposure–response coefficient (Vardoulakis et al. 2014). Numbers given in Table 4.

For our assessment of the impact of cool roofs, in summer we estimate that they could offset 17 deaths in 2006 and 34 in 2080s, a doubling of the beneficial effect, while the effect in winter remains moderate and remarkably constant for climate projections, with 4 additional deaths due to the impact of cool roofs in current and future climate (Table 4, Fig. 4). Therefore, we suggest that the overall impact of cool roofs on temperature-related mortality remains net beneficial in future for this region. It should be noted that this does not account for demographic changes, and daily mortality counts were held constant. Previously, estimates showed that heat-related mortality due to the UHI (difference between the red and green bars in Fig. 4) would more than double (from 96 in 2006, to 221 in 2080s), whereas cold-related mortality avoided by the UHI would change less (266 avoided in 2009, to 280 by 2080s) (Macintyre et al., 2021).

## 4 Discussion and conclusions

In this study we looked at the impact of cool roofs on the winter UHI, and cold-related mortality in winter. We found that the impact of cool roofs on the winter UHI, and subsequently on cold-related mortality is negligible, and thus supports implementing cool roofs as a strategy to reduce heat-related mortality, without weakening the protective effect of the winter UHI (Fig. 2, Fig. 3). In winter in the UK, days are shorter and sun angles lower, leading to less solar insolation, and other sources of heat dominating the UHI, such as anthropogenic and space heating sources. The low sun angles also account for the lower impact of cool roofs on ambient temperature during this winter period (Fig. 2, Table 2). The BEP scheme dynamically captures the effect of heat from buildings, and changes as the indoor-outdoor temperature gradient varies. However, the BEP model does not include an explicit anthropogenic heat flux, which is a limitation of our study. Anthropogenic sources of heat are challenging to quantify, and future work should include attempts to better approximate anthropogenic heat using assumptions about activity patterns, though detailed data is challenging to obtain (Hamilton et al., 2009), and similar modelling studies have shown that UHI intensification during particularly cold periods is independent of the background anthropogenic heat from sources other than buildings (such as vehicles) (Yang and Bou-Zeid, 2018).

Modelling tools such as WRF are useful for simulating interventions at a city or regional scale, where practical experiments would be costly and time consuming to implement at scale. The multi-layer urban canopy scheme (BEP) captures the interaction of buildings and roads on the lower atmosphere at sub-grid scale, including shading and reflections from buildings, and the model comparison is good (R^2^ > 0.9), though some very cold periods showed less agreement, possibly due to the representation of lying snow or frozen ground in the land surface scheme. While the WRF model has been used to study interventions and the UHI in summer, studies considering the winter are limited. Our finding of a −0.08 °C mean daytime population weighted change in exposure across the whole winter due to cool roofs, with up to −0.5 °C and occasionally −0.7 °C, is smaller than other studies that use the WRF model (He et al., 2020, Yang and Bou-Zeid, 2018), though these studies use a single layer urban canopy model (UCM) as opposed to the multilayer BEP used here, and there are differences in model resolution and choice of albedo change (He et al., 2020). Additionally, these studies are based in US cities at lower latitudes than the UK, so it may be that the lower solar insolation and even shorter day length in UK cities leads to a smaller impact of cool roofs on winter temperatures. Differences in sun angle and day length could also lead to our results for winter being about an order of magnitude smaller than results based on summer months (Macintyre and Heaviside, 2019).

Previous work showed that the winter UHI may protect against 266 deaths (2009/2010), and the summer UHI was associated with 96 additional deaths. However, looking to a future climate projection, heat-related mortality is expected to increase overall, and the number associated with the summer UHI will increase, with little reduction in mortality in winter (Hajat et al., 2014, Macintyre et al., 2021). This supports the case for focusing on interventions to reduce summer heat, while not worsening cold impacts in winter. We found that the impact of cool roofs on winter-time cold-related mortality is negligible, and thus supports implementing cool roofs as a strategy to reduce heat-related mortality (Fig. 3, Table 3). This effect also appears to be the case for future climate scenarios using a sensitivity study for projected future changes in temperature (Fig. 3), however we acknowledge that other factors that influence temperature-related mortality (such as population ageing, mortality counts, and behavioural adaptation) are not accounted for in this study, but should be included in future work. We note that for a milder winter, the protective effect of the UHI may be less pronounced, and as such milder winters should also be considered.

**Table 3:** Estimated cold-related mortality for the winter period (12 December 2009–28 February 2010). Numbers in brackets represent the 95% confidence intervals based on the exposure–response coefficients*.

Age group	Estimated total number of cold-related deaths (12 Dec 2009–28 Feb 2010)		
	URBAN	COOLROOF	RURAL
	(95% CI)	(95% CI)	(95% CI)
Total	**1743**	**1747**	**2009**
	(1511–1969)	(1515–1974)	(1745–2265)
0–64 yrs	**119**	**119**	**138**
	(27–206)	(27–207)	(32–239)
65–74 yrs	**249**	**249**	**284**
	(174–319)	(174–320)	(200–364)
75–84 yrs	**519**	**520**	**594**
	(403–630)	(404–631)	(463–719)
85 + yrs	**1041**	**1044**	**1182**
	(909–1169)	(912–1171)	(1034–1322)

* Exposure-response relationship used from Vardoulakis et al. (2014) for the West Midlands region: RR 1.8% (CI: 1.6%–2.1%) increase in mortality for every 1 °C decrease in daily mean ambient temperature below 11.7 °C (below 60th centile). Age graded coefficients; 0.7% (0–64 years), 1.6% (65–74 years), 1.8% (75–84 years), 3.1% (85 + years).

**Table 4:** Estimated cold- and heat-related mortality for the winter and summer periods in the context of climate change. Numbers in brackets represent the 95% confidence intervals based on the exposure–response coefficients (Vardoulakis et al., 2014). Numbers plotted in Fig. 4.

**Winter**				**Summer**			
	**URBAN**	**COOLROOF**	**RURAL**		**URBAN**	**COOLROOF**	**RURAL**
**2009/10**	**1743**	**1747**	**2009**	**2006**	**267**	**250**	**171**
	(1511–1969)	(1515–1974)	(1745–2265)		(218–315)	(204–295)	(139–202)
**2050s**	**1450**	**1454**	**1723**	**2050s**	**521**	**494**	**364**
	(1255–1641)	(1258–1646)	(1494–1947)		(426–613)	(404–581)	(297–429)
**2080s**	**1202**	**1206**	**1483**	**2080s**	**992**	**959**	**771**
	(1039–1363)	(1042–1368)	(1283–1677)		(814–1166)	(786–1126)	(631–907)

As with effects from heat, there is a clear increase in cold-related mortality with population age (Fig. 3, Table 3), which is important when targeting interventions aimed at reducing temperature-related harm to health. Our HIA is based on time series regression modelling of ambient temperature and mortality which assumes a fixed threshold for temperature, and we use region-specific exposure-response coefficients for heat and cold effects from the same study (Vardoulakis et al., 2014). The epidemiological study is based on observed temperatures, and we applied these results to temperatures generated by a high-resolution regional weather model; using different data sources could introduce additional uncertainty, though our model is evaluated against station observations, and the epidemiology is based on a gridded temperature dataset at the same resolution as our modelling. Cold-exposure is associated with mortality over the course of a few weeks (0–27 day lag), which makes it challenging to identify health effects and infer causality, compared with heat effects which typically occur within one or two days (Ryti et al., 2016). In this study the threshold for health effects from cold-exposure was assumed at the 60th centile of the annual temperature distribution (daily means), which broadly corresponds to the highest temperature in the coldest months of the year (December-March), meaning that a large number of days fall within this range. Cold thresholds are often hard to define, as increased risk of cold-related death can occur throughout much of the year (as days with ambient temperatures below the threshold are common), and we acknowledge that different temperature-mortality coefficients and thresholds would yield different results (Arbuthnott et al., 2018). There is little evidence for cold-wave effects as with heatwaves (Barnett et al., 2012), and also inconsistency in what is defined as a cold spell (Ryti et al., 2016).

Our analysis considers ambient temperatures, as exposure-response relationships derived for health effects are based on observed outdoor temperatures. Cool roofs may also have impacts on indoor temperatures, where people spend most of their time, and this may influence energy consumption within buildings (Taylor et al., 2018, Virk et al., 2014). Building retrofit for energy efficiency (such as cool roofs, insulation, and draught reduction) is a priority for governments to reduce greenhouse gas emissions^2^. With improving energy efficiency in buildings, homes should in theory be warmer in winter, use less energy, and people inside would be less exposed to cold, and thus a benefit for cold-related health effects. Meanwhile, the reduced energy use from insulated buildings would imply a smaller contribution to the winter UHI from space heating in buildings leaking to outdoors, which could theoretically reduce the winter UHI. However, for homes that are not able to improve insulation and energy efficiency, any reduction in the winter UHI may be problematic, and cold effects on health may become worse for those homes not able to improve insulation. Policy aimed at energy efficiency should therefore carefully consider the interlinked effects on health throughout the year, to identify potential issues that may be addressed with careful design, and due to the lifetime of such interventions, future climate projections should be considered where possible.

We show that a roofing intervention (reflective ‘cool’ roofs) aimed at reducing summer warming has an insignificant impact on the protective effect of the winter UHI, suggesting that this type of intervention may be useful for reducing heat-related mortality in summer (offsetting 17 deaths, 18% of heat-related mortality associated with the UHI) with little impact in wintertime (4 deaths, less than 2% of cold-related mortality). Using a sensitivity study for future climate, we show that the beneficial impact of cool roofs increases from avoiding 17 heat-related deaths in 2006, to 34 by the 2080s (RCP8.5), with no change in the impact of cool-roofs on cold-related mortality. These results further support cool roof interventions as a strategy to reduce ambient temperatures and reduce heat-related mortality, now and in future.

## Author contributions

HM, CH designed the study, HM performed the computational modelling and analysis work with input from CH and XMC, HM created figures and tables, all authors provided scientific feedback and helped shape and write the manuscript.

## Declaration of Competing Interest

The authors declare that they have no known competing financial interests or personal relationships that could have appeared to influence the work reported in this paper.

## Footnotes

1 Output Areas (OA) are the lowest geographical level at which census estimates are provided. There are a total of 181,408 Output Areas in England and Wales, with an average population of 309 per OA.

2 In June 2019 the UK Government became the first globally to pass a net zero emissions target into law; Climate Change Act 2008 (2050 Target Amendment) Order 2019 http://www.legislation.gov.uk/uksi/2019/1056/contents/made.

## References

[b0005] Arbuthnott K., Hajat S., Heaviside C., Vardoulakis S. (2016). Changes in population susceptibility to heat and cold over time: assessing adaptation to climate change. Environ. Health.

[b0010] Arbuthnott K., Hajat S., Heaviside C., Vardoulakis S. (2018). What is cold-related mortality? A multi-disciplinary perspective to inform climate change impact assessments. Environ. Int..

[b0015] Barnett A.G., Hajat S., Gasparrini A., Rocklöv J. (2012). Cold and heat waves in the United States. Environ. Res..

[b0020] Basu R. (2009). High ambient temperature and mortality: a review of epidemiologic studies from 2001 to 2008. Environ. Health: Global Access Sci. Source.

[b0025] Besir A.B., Cuce E. (2018). Green roofs and facades: A comprehensive review. Renew. Sustain. Energy Rev..

[b0030] Chen F., Kusaka H., Bornstein R., Ching J., Grimmond C.S.B., Grossman-Clarke S., Loridan T., Manning K.M., Martilli A., Miao S., Sailor D., Salamanca F.P., Taha H., Tewari M., Wang X., Wyszogrodzki A.A., Zhang C. (2011). The integrated WRF/urban modelling system: development, evaluation, and applications to urban environmental problems. Int. J. Climatol..

[b0035] Gedzelman S.D., Austin S., Cermak R., Stefano N., Partridge S., Quesenberry S., Robinson D.A. (2003). Mesoscale aspects of the Urban Heat Island around New York City. Theor. Appl. Climatol..

[b0040] Gómez-Acebo I., Llorca J., Dierssen T. (2013). Cold-related mortality due to cardiovascular diseases, respiratory diseases and cancer: a case-crossover study. Public Health.

[b0045] Guo Y., Gasparrini A., Armstrong B., Li S., Tawatsupa B., Tobias A. (2014). Global variation in the effects of ambient temperature on mortality: a systematic evaluation. Epidemiology.

[b0050] Hajat S., Vardoulakis S., Heaviside C., Eggen B. (2014). Climate change effects on human health: Projections of temperature-related mortality for the UK during the 2020s, 2050s and 2080s. J. Epidemiol. Commun. Health.

[b0055] Hamilton I.G., Davies M., Steadman P., Stone A., Ridley I., Evans S. (2009). The significance of the anthropogenic heat emissions of London’s buildings: A comparison against captured shortwave solar radiation. Build. Environ..

[b0060] He C., He L., Zhang Y., Kinney P.L., Ma W. (2020). Potential impacts of cool and green roofs on temperature-related mortality in the Greater Boston region. Environ. Res. Lett..

[b0065] Heaviside C., Macintyre H.L., Vardoulakis S. (2017). The Urban Heat Island: Implications for health in a changing environment. Curr. Environ. Health Rep..

[b0070] Heaviside C., Vardoulakis S., Cai X. (2016). Attribution of mortality to the Urban Heat Island during heatwaves in the West Midlands, UK. Environ. Health.

[b0075] HPA (2012). Health Effects of Climate Change in the UK 2012.

[b0080] Imhoff M.L., Zhang P., Wolfe R.E., Bounoua L. (2010). Remote sensing of the urban heat island effect across biomes in the continental USA. Remote Sens. Environ..

[b0085] Kershaw T., Sanderson M., Coley D., Eames M. (2010). Estimation of the urban heat island for UK climate change projections. Build. Serv. Eng. Res. Technol..

[b0090] Kłysik K., Fortuniak K. (1999). Temporal and spatial characteristics of the urban heat island of Łódź, Poland. Atmosph. Environ..

[b0095] Kolokotroni M., Gowreesunker B.L., Giridharan R. (2013). Cool roof technology in London: An experimental and modelling study. Energy Build..

[b0100] Kolokotroni M., Ren X., Davies M., Mavrogianni A. (2012). London’s urban heat island: Impact on current and future energy consumption in office buildings. Energy Build..

[b0105] Li D., Bou-Zeid E., Oppenheimer M. (2014). The effectiveness of cool and green roofs as urban heat island mitigation strategies. Environ. Res. Lett..

[b0110] Lowe, J.A., Bernie, D., Bett, P., Bricheno, L., Brown, S., Calvert, D., Clark, R., Eagle, K., Edwards, T., Fosser, G., Fung, F., Gohar, L., Good, P., Gregory, J., Harris, G., Howard, T., Kaye, N., Kendon, E., Krijnen, J., Maisey, P., McDonald, R., McInnes, R., McSweeney, C., Mitchell, J.F.B., Murphy, J., Palmer, M., Roberts, C., Rostron, J., Sexton, D., Thornton, H., Tinker, J., Tucker, S., Yamazaki, K., Belcher, S., 2018. UKCP18 Science Overview Report. Met Office Hadley Centre, Exeter. http://ukclimateprojections.metoffice.gov.uk/.

[b0115] Macintyre H.L., Heaviside C. (2019). Potential benefits of cool roofs in reducing heat-related mortality during heatwaves in a European city. Environ. Int..

[bib241] Macintyre H.L., Heaviside C., Cai X., Phalkey R. (2021). The winter urban heat island: Impacts on cold-related mortality in a highly urbanized European region for present and future climate. Environ. Int..

[b0120] Macintyre H.L., Heaviside C., Taylor J., Picetti R., Symonds P., Cai X.M., Vardoulakis S. (2018). Assessing urban population vulnerability and environmental risks across an urban area during heatwaves – Implications for health protection. Sci. Total Environ..

[b0130] Martilli A., Clappier A., Rotach M. (2002). An Urban Surface Exchange Parameterisation for Mesoscale Models. Boundary-Layer Meteorol..

[b0135] Met Office, 2012. Met Office Integrated Data Archive System (MIDAS) Land and Marine Surface Stations Data (1853-current). NCAS British Atmospheric Data Centre. http://catalogue.ceda.ac.uk/uuid/220a65615218d5c9cc9e4785a3234bd0.

[b0140] Milojevic A., Armstrong B.G., Gasparrini A., Bohnenstengel S.I., Barratt B., Wilkinson P. (2016). Methods to Estimate Acclimatization to Urban Heat Island Effects on Heat- and Cold-Related Mortality. Environ. Health Perspect..

[b0145] Morini E., Touchaei A., Castellani B., Rossi F., Cotana F. (2016). The Impact of Albedo Increase to Mitigate the Urban Heat Island in Terni (Italy) Using the WRF Model. Sustainability.

[b0155] Oleson K.W., Bonan G.B., Feddema J. (2010). Effects of white roofs on urban temperature in a global climate model. Climate.

[b0160] ONS, 2011. Office for National Statistics, Census.

[b0150] Morini E., Touchaei A.G., Rossi F., Cotana F., Akbari H. (2018). Evaluation of albedo enhancement to mitigate impacts of urban heat island in Rome (Italy) using WRF meteorological model. Urban Clim..

[b0170] Runnalls K.E., Oke T.R. (2000). Dynamics and controls of the near-surface heat island of Vancouver, British Columbia. Phys. Geography.

[b0165] ONS, 2015. Office for National Statistics. Annual Mid-year Population Estimates, 2014.

[b0175] Ryti N.R.I., Guo Y., Jaakkola J.J.K. (2016). Global association of cold spells and adverse health effects: a systematic review and meta-analysis. Environ. Health. Perspect..

[b0180] Santamouris M. (2015). Analyzing the heat island magnitude and characteristics in one hundred Asian and Australian cities and regions. Sci. Total Environ..

[b0185] Silva H.R., Phelan P.E., Golden J.S. (2010). Modeling effects of urban heat island mitigation strategies on heat-related morbidity: a case study for Phoenix, Arizona, USA. Int J Biometeorol.

[b0190] Smith K.R., Roebber P.J. (2011). Green Roof Mitigation Potential for a Proxy Future Climate Scenario in Chicago, Illinois. J. Appl. Meteorol. Climatol..

[b0195] Stone B., Vargo J., Liu P., Habeeb D., DeLucia A., Trail M., Hu Y., Russell A. (2014). Avoided Heat-Related Mortality through Climate Adaptation Strategies in Three US Cities. PLoS ONE.

[b0200] Susca T. (2012). Multiscale Approach to Life Cycle Assessment. J. Ind. Ecol..

[b0205] Taylor J., Symonds P., Wilkinson P., Heaviside C., Macintyre H.L., Davies M., Mavrogianni A., Hutchinson E. (2018). Estimating the Influence of Housing Energy Efficiency and Overheating Adaptations on Heat-Related Mortality in the West Midlands, UK. Atmosphere.

[b0210] U.S. Environmental Protection Agency, 2008. Reducing Urban Heat Islands: Compendium of Strategies. United States Environmental Protection Agency. http://www.epa.gov/heat-islands/heat-island-compendium.

[b0215] Vahmani P., Sun F., Hall A., Ban-Weiss G. (2016). Investigating the climate impacts of urbanization and the potential for cool roofs to counter future climate change in Southern California. Environ. Res. Lett..

[b0220] van Ruijven B.J., De Cian E., Sue Wing I. (2019). Amplification of future energy demand growth due to climate change. Nat. Commun..

[b0225] Vardoulakis S., Dear K., Hajat S., Heaviside C., Eggen B., McMichael A. (2014). Comparative assessment of the effects of climate change on heat- and cold-related mortality in the United Kingdom and Australia. Environ. Health Perspect..

[b0230] Virk G., Jansz A., Mavrogianni A., Mylona A., Stocker J., Davies M. (2014). The effectiveness of retrofitted green and cool roofs at reducing overheating in a naturally ventilated office in London: Direct and indirect effects in current and future climates. Indoor Built Environ..

[b0235] Virk G., Jansz A., Mavrogianni A., Mylona A., Stocker J., Davies M. (2015). Microclimatic effects of green and cool roofs in London and their impacts on energy use for a typical office building. Energy Build..

[b0240] Yang J., Bou-Zeid E. (2018). Should Cities Embrace Their Heat Islands as Shields from Extreme Cold?. J. Appl. Meteorol. Climatol..

