# Supplementary material for: Comparing temperature-related mortality impacts of cool roofs in winter and summer in a highly urbanized European region for present and future climate
Source: Environ Int. 2021 Sep;154:106606. doi: 10.1016/j.envint.2021.106606 (PMC8214226; doi:10.1016/j.envint.2021.106606)
Supplement: Supplementary Data 1 [file mmc1.docx]

**Supplementary Information**

Supplementary information for “Comparing temperature-related mortality impacts of cool roofs in winter and summer in a highly urbanized European region for present and future climate”

**Detailed modelling set up and evaluation**

We used the WRF model version 3.6.1 (Chen et al. 2011) with four nested domains, adopting horizontal grid resolutions of 36 km, 12 km, 3 km, and 1 km, respectively, with two-way feedback between grids (Fig.1). The model time-steps in each domain were 180, 60, 15 and 5 seconds, respectively, whilst meteorological variables, including 2 metre air temperature were output at hourly intervals. Boundary conditions for the outermost domain were from the ERA-Interim reanalysis at 0.5° every 6 hours (Dee et al. 2011), and there were 39 pressure levels above the surface, up to 1 hPa. We used a multilayer urban canopy scheme called Building Energy Parameterisation (BEP), which models the effect of buildings on horizontal and vertical energy and momentum fluxes inside and immediately above the urban street canyons, at a vertical resolution of 5 m, accounting for shading and radiation trapping in street canyons (Martilli et al. 2002). Information on building and road properties (e.g. building height, street canyon width, material properties such as albedo, thermal conductivity and heat capacity) is prescribed for three urban categories: Industrial/commercial, High-intensity residential, and Low-intensity residential, across the West Midlands region (Fig.1b) (Heaviside et al. 2015). The BEP scheme assumes a constant internal building temperature of 20°C, which adds to the ground heat flux in the model through thermal conduction through the walls and roof; this does not explicitly consider other anthropogenic heat fluxes (such as from vehicles) during simulations. Land-surface data used as input to WRF for all domains were based on the US Geological Survey (USGS) 24-category land-use data, and for the inner domain we used two local datasets to generate the three separate urban categories (Owen et al. 2006). We used the Noah Land Surface Model (Noah-LSM), which is often coupled with an urban canopy scheme, and has four layers of soil moisture and soil temperature (Tewari et al. 2004). A tiled approach based on the urban fraction in each grid cell is used to divide energy fluxes between the BEP (urban) and Noah-LSM. The model has been previously run and validated for other periods using a similar configuration (Macintyre and Heaviside 2019; Macintyre et al. 2018).

| **Table S1.** (a) Details of default urban categories used in BEP (Building Energy Parameterisation). (b) General WRF model set-up details. | | | | |
| --- | --- | --- | --- | --- |
| **(a)** | |  |  |  |
| **Category** | | **1: Industrial/ commercial** | **2: High-intensity residential** | **3: Low-intensity residential** |
| Albedo (roof, wall, ground) | | 0.1989 | 0.1997 | 0.2027 |
| Surface emissivity (roof, wall, ground) | | 0.9239 | 0.9274 | 0.9292 |
| Average building height | | 25 m | 15 m | 10 m |
| **(b)** |  | | |  |
| **Model setting** | **Option** | | | **Reference** |
| Long wave radiation | Rapid Radiative Transfer Model (RRTM) | | | Mlawer et al. (1997) |
| Short-wave radiation | Dudhia scheme | | | Dudhia (1989) |
| Boundary layer physics | Bougeault–Lacarrere (designed for use with BEP urban scheme) | | | Bougeault and Lacarrere (1989) |
| Urban physics | BEP urban scheme | | | Martilli et al. (2002) |

| **Table S2.** Model evaluation of 2 m temperature for the seasonal urban simulation based on MIDAS meteorological station observations^*^. | | | | | | | | |
| --- | --- | --- | --- | --- | --- | --- | --- | --- |
|  | Edgbaston (EB) | | Coventry (CV) | | Coleshill (CH) | | Winterbourne (WB) | |
|  | Observed | Modelled | Observed | Modelled | Observed | Modelled | Observed | Modelled |
| Mean (°C) | 3.30 | 3.47 | 3.28 | 3.28 | 3.24 | 2.89 | 3.17 | 3.27 |
| Standard deviation (°C) | 3.63 | 3.63 | 3.80 | 3.90 | 3.94 | 4.08 | 3.74 | 3.70 |
| RMSD^‡^ (°C) | – | 1.41 | – | 1.62 | – | 1.85 | – | 1.69 |
| Correlation coefficient | – | 0.93 | – | 0.91 | – | 0.90 | – | 0.90 |
| *Modelled data extracted by bilinear interpolation from temperatures at the nearest four grid points in the 1 km grid spacing. Numbers are calculated across all hourly values for the modelled period (15 Nov 09 – 28 Feb 10). In this context, standard deviation is indicative of the diurnal cycle across the modelled period.  ‡RMSD is the root mean square deviation, calculated from hourly values, as follows: $RMSD=\sqrt{\frac{\sum_{n=1}^{N} \left( \text{model}_{n}-\text{observed}_{n} \right)^{2}}{N}}$ | | | | | | | | |

| 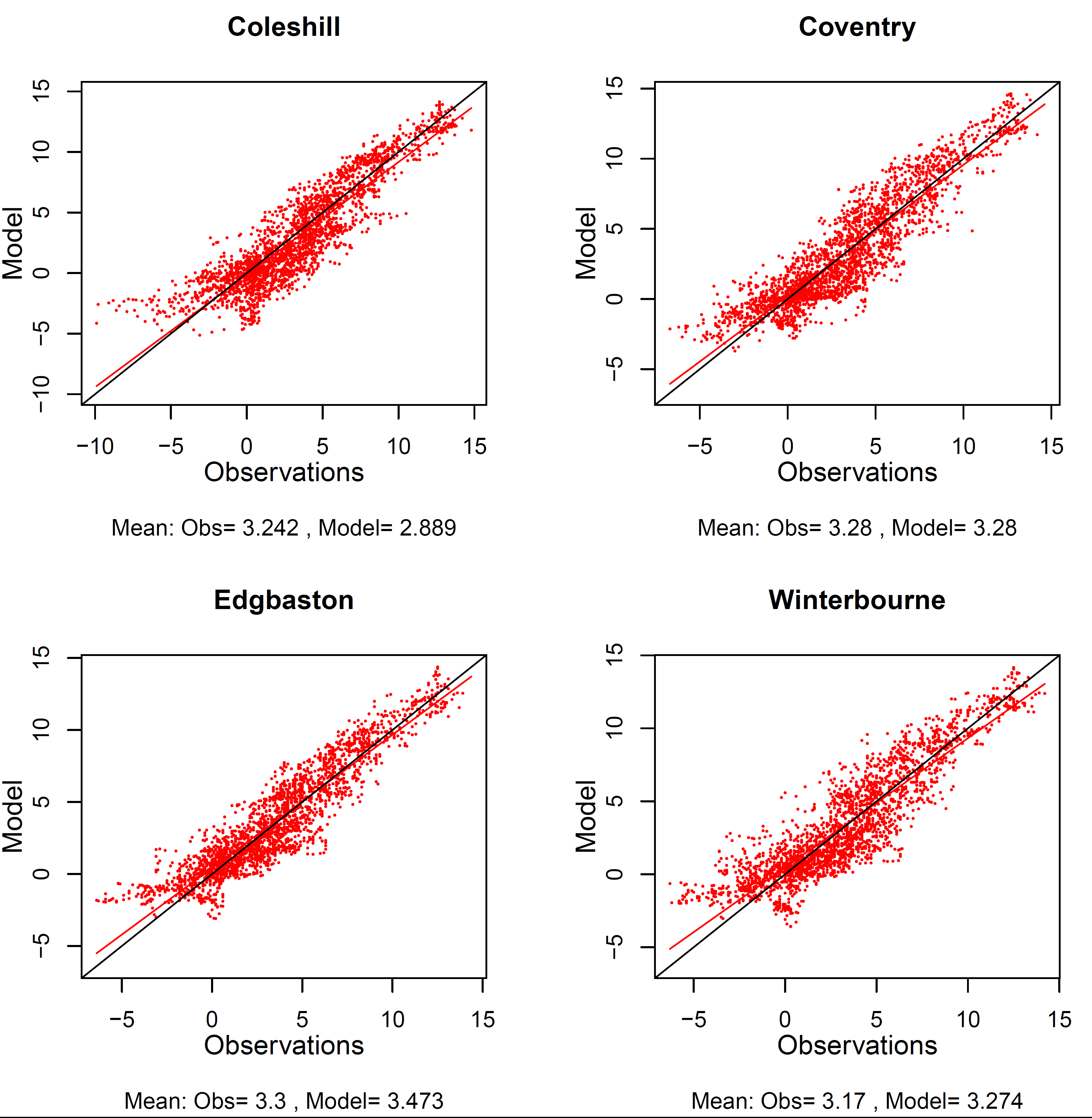 |
| --- |
| Fig. S1. Scatter plots of modelled vs observed hourly 2 m air temperature. |

|  |
| --- |
| Fig. S2. Taylor plot for observed vs modelled values for the ‘urban’ WRF simulation. |

**References**

Bougeault, P.; Lacarrere, P. Parameterization of Orography-Induced Turbulence in a Mesobeta--Scale Model. Monthly Weather Review 1989;117:1872-1890

Chen, F.; Kusaka, H.; Bornstein, R.; Ching, J.; Grimmond, C.S.B.; Grossman-Clarke, S.; Loridan, T.; Manning, K.M.; Martilli, A.; Miao, S.; Sailor, D.; Salamanca, F.P.; Taha, H.; Tewari, M.; Wang, X.; Wyszogrodzki, A.A.; Zhang, C. The integrated WRF/urban modelling system: development, evaluation, and applications to urban environmental problems. International Journal of Climatology 2011;31:273-288

Dee, D.P.; Uppala, S.M.; Simmons, A.J.; Berrisford, P.; Poli, P.; Kobayashi, S.; Andrae, U.; Balmaseda, M.A.; Balsamo, G.; Bauer, P.; Bechtold, P.; Beljaars, A.C.M.; van de Berg, L.; Bidlot, J.; Bormann, N.; Delsol, C.; Dragani, R.; Fuentes, M.; Geer, A.J.; Haimberger, L.; Healy, S.B.; Hersbach, H.; Hólm, E.V.; Isaksen, L.; Kållberg, P.; Köhler, M.; Matricardi, M.; McNally, A.P.; Monge-Sanz, B.M.; Morcrette, J.J.; Park, B.K.; Peubey, C.; de Rosnay, P.; Tavolato, C.; Thépaut, J.N.; Vitart, F. The ERA-Interim reanalysis: configuration and performance of the data assimilation system. Quarterly Journal of the Royal Meteorological Society 2011;137:553-597

Dudhia, J. Numerical Study of Convection Observed during the Winter Monsoon Experiment Using a Mesoscale Two-Dimensional Model. Journal of the Atmospheric Sciences 1989;46:3077-3107

Heaviside, C.; Cai, X.-M.; Vardoulakis, S. The effects of horizontal advection on the urban heat island in Birmingham and the West Midlands, United Kingdom during a heatwave. Quarterly Journal of the Royal Meteorological Society 2015;141:1429-1441

Macintyre, H.L.; Heaviside, C. Potential benefits of cool roofs in reducing heat-related mortality during heatwaves in a European city. Environment International 2019;127:430-441

Macintyre, H.L.; Heaviside, C.; Taylor, J.; Picetti, R.; Symonds, P.; Cai, X.M.; Vardoulakis, S. Assessing urban population vulnerability and environmental risks across an urban area during heatwaves – Implications for health protection. Science of The Total Environment 2018;610–611:678-690

Martilli, A.; Clappier, A.; Rotach, M. An Urban Surface Exchange Parameterisation for Mesoscale Models. Boundary-Layer Meteorol 2002;104:261-304

Mlawer, E.J.; Taubman, S.J.; Brown, P.D.; Iacono, M.J.; Clough, S.A. Radiative transfer for inhomogeneous atmospheres: RRTM, a validated correlated-k model for the longwave. Journal of Geophysical Research: Atmospheres 1997;102:16663-16682

Owen, S.M.; MacKenzie, A.R.; Bunce, R.G.H.; Stewart, H.E.; Donovan, R.G.; Stark, G.; Hewitt, C.N. Urban land classification and its uncertainties using principal component and cluster analyses: A case study for the UK West Midlands. Landscape and Urban Planning 2006;78:311-321

Tewari, M.; Chen, F.; Wang, W.; Dudhia, J.; LeMone, M.A.; Mitchell, K.; Ek, M.; Gayno, G.; Wegiel, J.; Cuenca, R.H. Implementation and verification of the unified Noah land surface model in the WRF model. 20th Conference on Weather Analysis and Forecasting/16th Conference on Numerical Weather Prediction. Seattle, Washington.; 2004
